# National common metrics for the NIH Clinical and Translational Science Award Institutions: A signal of the transformation of the American biomedical research enterprise

**DOI:** 10.1017/cts.2019.430

**Published:** 2019-10-08

**Authors:** Harry P. Selker

**Affiliations:** 1 Institute for Clinical Research and Health Policy Studies, Tufts Medical Center, Boston, MA, USA; 2 Tufts Clinical and Translational Science Institute, Tufts University, Medford, MA, USA; 3 Clinical Research Forum, Washington, DC, USA

**Keywords:** Clinical and translational science, research process improvement, common metrics, biomedical research enterprise, National Center for Advancing Translational Science

In 2006, the National Institutes of Health (NIH) initiated the Clinical and Translational Science Award (CTSA) program with the auspicious aim of transforming the American biomedical research enterprise [1]. The spectacular advances in medical research seen in preceding decades were translating more slowly than wanted into widespread patient care and public health. Needed, it was sensed, was a new fierce focus on translation. A big bet was made: The national CTSA consortium hubs were created at leading US research institutions that had three mandates: (1) to provide excellent core research resources, services, mentoring, and training, (2) to develop novel research methods, and (3) to reengineer the nation’s clinical and translational research enterprise. To facilitate this, in 2011, NIH established the National Center for Advancing Translational Sciences (NCATS) as the new home for the CTSA Program and as a laboratory for innovations and accelerated progress in translational science.

Reflecting keen interest in the CTSA mission, in 2011, Congress asked the Institute of Medicine (IOM) to report on the performance and opportunities of the CTSA consortium. Among its recommendations, issued in 2013, the IOM committee urged NCATS to create CTSA consortium-wide evaluation and improvement processes, including shared *common metrics* of effectiveness and impact [2]. Such metrics were proposed to enhance transparency, accountability, continuous improvement, and strategic management of the overall CTSA consortium and its individual hubs. As a major priority, NCATS undertook a collaborative process across the CTSA consortium, the Common Metrics Initiative.

In 2015, to start the Common Metrics Initiative, NCATS and CTSA consortium leaders and staff formed workgroups to formulate common metrics. Ultimately, three metrics were chosen for initial implementation: (1) median time duration of institutional review board review, as a way to assess a component of the efficiency of clinical trial execution; (2) research career outcomes of CTSA trainees and mentees, to assess CTSA career development programs; and (3) the generation of publications and follow-on funding from pilot projects, to measure the impact of CTSA pilot awards.

After iterative revision by the workgroups, in 2016, 64 CTSA hub institutions began using the 3 common metrics as the first national implementation of common performance metrics and process improvement across an NIH national research consortium. Led by Tufts Clinical and Translational Science Institute (CTSI), the initial implementation and evaluation of this effort are described in a report to NCATS and are published elsewhere [3, 4]. A review of those reports will make clear that the institution of the common metrics was not easy. Marshalling the effort, resources, and needed engagement at CTSA institutions was very challenging. Also, there were concerns about the meaningfulness of the first metrics that were implemented. It was understood that this was a first (major) effort, and that improvements and enhancements will be needed. Yet already, the metrics have catalyzed conversations and plans at hubs and across the consortium that have led to self-assessment and process improvements [3, 4]. The program is now poised for expansion and refinement by a CTSA Coordinating Center for common metrics and related activities. Thereby, the program is beginning to fulfill its purpose of improving the conduct of translational research nationally.

In introducing the metrics, it was made clear that while all CTSA hubs should be using the same metrics in common, but not all hubs should be doing the same thing. When aggregated, the common metrics will provide a valuable overarching description of the CTSA consortium’s activities, but the primary purpose is to help *individual hubs* improve performance in their own targeted areas. This is consistent with NCATS’ expectation that CTSA institutions not aspire to cover all possible areas, but rather, to build on and leverage their own special strengths and circumstances for the translational mission. The net national benefit will be greatest if individual hubs each make their own best contributions. This requires institutions to carefully and candidly assess their areas of research and academic excellence to determine which are most suitable for leverage and/or development. The use of the common metrics, and importantly, other metrics targeted at their own needs, will help advance this.

Additionally, the use of metrics can help CTSAs in their avowed intent to make innovations in research processes. CTSAs are being pushed to reexamine long-held practices and to be open to, and committed to, changing the way research is done. For example, in traditional project-focused research, processes and planned outcomes are not evaluated, not held accountable to their original aims, nor improved based on performance. Metrics can be an important part of transformation of the research enterprise that will propel translation. They can help assess the sufficiency of a shift beyond grant-funded projects that generate publications but do not necessarily meet originally stated objectives. Instead, CTSAs encourage ongoing evaluation of the processes of research, the support processes that are optimal, and that objectives are met on time.

As another example, generally successful research labs and projects have focused on small components of the overall translational chain. Researchers have not always started their work knowledgeable of, or even thinking about, alignment of all the links and steps needed to have ultimate impact on healthcare and the public’s health. Also, too often, projects have been limited to the specific scientific domain of the principal investigator rather than including the multiple disciplines, stakeholders, and communities that could greatly improve the project and its impact. Therefore, metrics must assess the incorporation of multiple disciplines, institutions, organizations, perspectives, and stakeholders [5].

In considering these and other examples, it must be remembered that metrics alone are meaningless, and will have no impact, unless they are specific and closely aligned to the ultimate aims. *They must be embedded in an improvement framework in which we are using them to assess progress toward achieving our aims in the research environment, operations, processes, and culture*.

Ultimately, to have impact, together, metrics and improvement efforts must serve the translational mission and needed cultural change, not just measure details. Metrics must reinforce having impact on health for individuals, communities, and healthcare systems. Drawing from biomedical, clinical, health services, policy fields, and many complementary disciplines, with whatever partners and in whatever settings, *each project must have a clear line of sight to impact on healthcare and/or population health*. This does *not* mean that basic and preclinical research are not valued – indeed they are critical. However, even at the basic level, for there to be successful translation to impact, that desired result must be part of the vision at the outset. In all biomedical research, at the outset, consideration must be given to what kind of activities, programs, and possible spin-off enterprises might best facilitate wider dissemination and impact. That translational vision will be powered and accelerated by measurement and process improvement.

Taken together, metrics of research processes and outcomes, including shared common metrics, are key tools only if they serve a mission. Ideally, they will improve our understanding and execution of “the science of doing science.” Targeted and used wisely, the national CTSA Common Metrics Initiative will help move us ahead on a path of transformation of the American biomedical research enterprise, for the benefit of all.
